# Radiolytic Formation of Fe_3_O_4_ Nanoparticles: Influence of Radiation Dose on Structure and Magnetic Properties

**DOI:** 10.1371/journal.pone.0090055

**Published:** 2014-03-07

**Authors:** Alam Abedini, Abdul Razak Daud, Muhammad Azmi Abdul Hamid, Norinsan Kamil Othman

**Affiliations:** School of Applied Physics, Faculty of Science and Technology, Universiti Kebangsaan Malaysia, Bangi, Selangor, Malaysia; RMIT University, Australia

## Abstract

Colloidal Fe_3_O_4_ nanoparticles were synthesized using a gamma-radiolysis method in an aqueous solution containing iron chloride in presence of polyvinyl alcohol and isopropanol as colloidal stabilizer and hydroxyl radical scavenger, respectively. Gamma irradiation was carried out in a ^60^Co gamma source chamber at different absorbed doses. Increasing the radiation dose above a certain critical dose (100 kGy) leads to particle agglomeration enhancement, and this can influence the structure and crystallinity, and consequently the magnetic properties of the resultant particles. The optimal condition for formation of Fe_3_O_4_ nanoparticles with a uniform and narrow size distribution occurred at a dose of 100 kGy, as confirmed by X-ray diffractometry and transmission electron microscopy. A vibrating sample magnetometry study showed that, when radiation dose increased, the saturation and remanence magnetization decreased, whereas the coercivity and the remanence ratio increased. This magnetic behavior results from variations in crystallinity, surface effects, and particle size effects, which are all dependent on the radiation dose. In addition, Fourier transform infrared spectroscopy was performed to investigate the nature of the bonds formed between the polymer chains and the metal surface at different radiation doses.

## Introduction

Magnetic nanoparticles have attracted considerable interest in recent years by virtue of their unique physical and chemical properties, which can differ significantly from the bulk or molecular properties of the respective materials [1]. Because of their proposed applications in several areas such as medical technologies, both in vivo and in vitro, much effort has been dedicated to the preparation of magnetic nanoparticles [2]. Magnetite (Fe_3_O_4_) nanoparticles in particular are considered to be highly promising candidates for a broad range of applications because of their unique structural and magnetic properties, non-toxicity, and high chemical stability [3], [4], [5], [6]. Magnetic nanoparticles for biomedical uses should possess certain physical features such as small size and a narrow size distribution in order to provide uniform physical and chemical properties in addition to superparamagnetic behavior. These properties make them an ideal candidate for applications such as targeted drug delivery [7], [8], [9], hyperthermic treatments [10], [11], [12], magnetic resonance imaging enhancement, and sensing devices [13], [14].

There are various wet chemical methods for preparing the magnetic nanoparticles such as solvothermal [15], [16], sol-gel [17], [18], thermal decomposition [19], [20], and coprecipitation [21], [22] techniques. In addition to these, radiolytic reduction is another promising technique [23], [24].

Typically, the radiation source used in radiolytic reduction is either an electron beam or gamma-ray generator. In gamma radiolysis, a large number of solvated electrons are produced during irradiation in an aqueous solution. These can reduce the metal ions to lower oxidation states or even to neutral metal atoms [25]. Furthermore, this technique avoids the use of reducing agents and the associated side reactions. The reduction of metal ions under gamma irradiation will lead to the formation of metal nanoparticles. In the common chemical reduction methods, the rate of the reducing reaction is the primary parameter determining the size of the nanoparticles, so that high reducing agent concentrations are preferable for small nanoparticles. In the radiolytic reduction method, the average size of the nanoparticles is influenced by the concentration of reducing species released during irradiation. This concentration can be controlled via the irradiation dose. Additionally, the reducing species generated by the radiation, penetrates deeply into the aqueous sample and randomly reduces the metal ions throughout the solution. Consequently, newly-formed nuclei will be distributed homogeneously throughout the solution, and this tends to produce highly dispersed nanoparticles [26].

This paper reports the synthesis of colloidal Fe_3_O_4_ nanoparticles using a radiolytic reduction method in an aqueous solution. The influence of radiation dose on the formation, structure, and magnetic properties of the Fe_3_O_4_ nanoparticles were studied and discussed.

## Experimental section

### Materials

Ferric chloride hexahydrate (FeCl_3_.6H_2_O), used as the precursor material, was purchased from Sigma Aldrich (Missouri, United States). Polyvinyl alcohol (PVA, molecular weight = 89,000, Sigma Aldrich), isopropanol, and sodium hydroxide (Na(OH), Macron, Pennsylvania, United States) were used as a stabilizer to control the growth, a hydroxyl radical scavenger, and a pH adjuster, respectively. Deionized water was used as the solvent.

### Synthesis Procedure

The magnetic nano-colloids based on magnetite (Fe_3_O_4_) and organic solvents were prepared using a gamma irradiation technique from an aqueous salt solution of Fe(III) in an alkaline medium and suspended in a carrier liquid. 2 mmol of FeCl_3_·6H_2_O was dissolved in a stock solution of 4% PVA, which was prepared by dissolving PVA powder in deionized water in presence of isopropanol. The solution was stirred for 1 h at 70°C, then 0.4 M Na(OH) was added dropwise to the solution to increase the pH to 12. This final solution was irradiated with gamma-rays from a ^60^Co source to various absorbed doses up to 200 kGy at a dose rate of 2 kGy/h.

### Characterization

The structural characteristics and average particle sizes were determined using transmission electron microscopy (TEM) on a Phillips CM-12 operated at 100 kV. Samples for TEM studies were prepared by placing a drop of the irradiated solutions on a copper TEM grid. Prior to microscopy, the samples were allowed to dry naturally on the grids for several hours. Fourier transform infrared spectroscopy (FT-IR) spectra were recorded in the range 650–4,000 cm^−1^ on a Spectrum 400 FTIR spectrophotometer (Perkin Elmer) with the samples embedded in KBr pellets. The crystal structure of the Fe_3_O_4_ nanoparticles was characterized using X-ray diffractometry (XRD), performed at a scanning rate of 0.025°/0.1 s over the 2θ range 20–90° on a D_8_ Advance diffractometer (Bruker) with graphite-monochromatized Cu-K_α_ radiation (λ = 1.5406 Å). The magnetic properties of nanoparticles were determined at 25°C using a model 9500 (LDJ) computerized vibrating sample magnetometer.

## Results and discussion

### Formation of PVA-coated Fe_3_O_4_ nanoparticles

**Figure 1:**
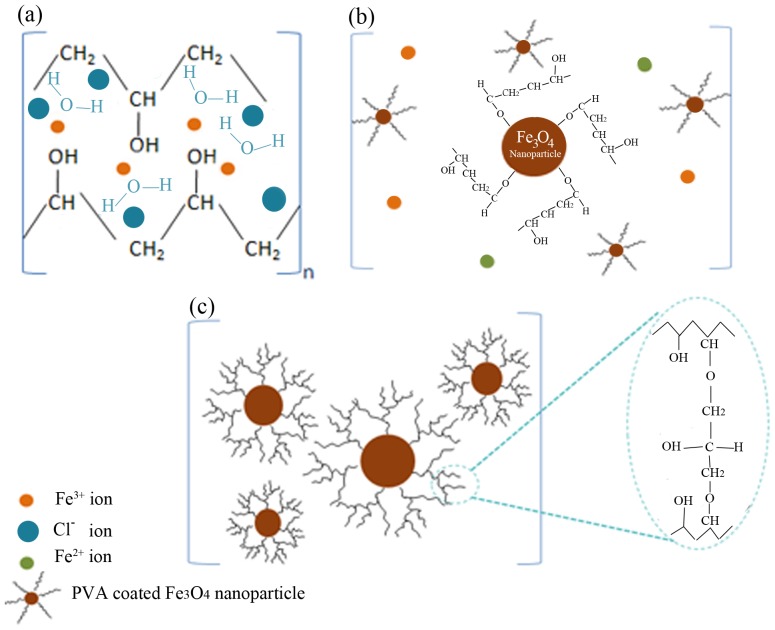
A proposed simplified mechanism of formation PVA-ion complexes and PVA-Fe_3_O_4_ nanoparticles. (**a**) interactions between PVA molecules and Fe^3+^ ions before irradiation; (**b**) interaction between PVA and the surfaces of Fe_3_O_4_ nanoparticles after irradiation; and (**c**) coating of the Fe_3_O_4_ nanoparticles by crosslinking of the polymer at high radiation doses.

A simplified scheme of the interactions between the PVA capping agent and the metal ions, both before and after irradiation, is shown in Figure 1. Prior to irradiation, the Fe (III) ions are bound by ionic bonds to the polymeric chains (Figure 1-a). PVA acts as a stabilizer for dissolved metallic salts through steric and electrostatic stabilization. However, the full mechanism is more complex, because of the presence of hydrogen bonds, both between the water molecules themselves and between the water molecules and the polarized groups on the polymer. When the system was irradiated by gamma rays, many active intermediates were generated during the radiolysis of water [25]. 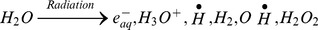
(1)Among these active species, the solvated electrons (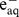) are strong reducing agents and can reduce Fe (III) ions into lower-state Fe (II) ions and, with very low probability, neutral iron atoms:
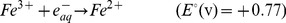
(2a)or
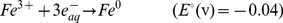
(2b)Because the system was kept in an oxidative air atmosphere during irradiation, these iron particles were quickly oxidized because of their high activity, resulting in the formation of Fe_3_O_4_ nanoparticles [27].
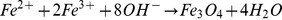
(3)The metal nanoparticle surfaces are likely to be stabilized through strong associations between the Fe_3_O_4_ nanoparticle surfaces and the ‘O’ atoms of the carboxyl groups of PVA (Figure 1-b) [28]. With increasing gamma dose, additional polymer radicals are formed indirectly by reaction with the H^•^ and OH^•^ radicals that arise from the irradiation of water molecules. These polymer radicals may interact with one another by disproportion, and combination through inter- and/or intra-molecular crosslinking, giving a 3D polymer network that can influence the crystal structure of Fe_3_O_4_ nanoparticles (Figure 1-c) [29].

**Figure 2:**
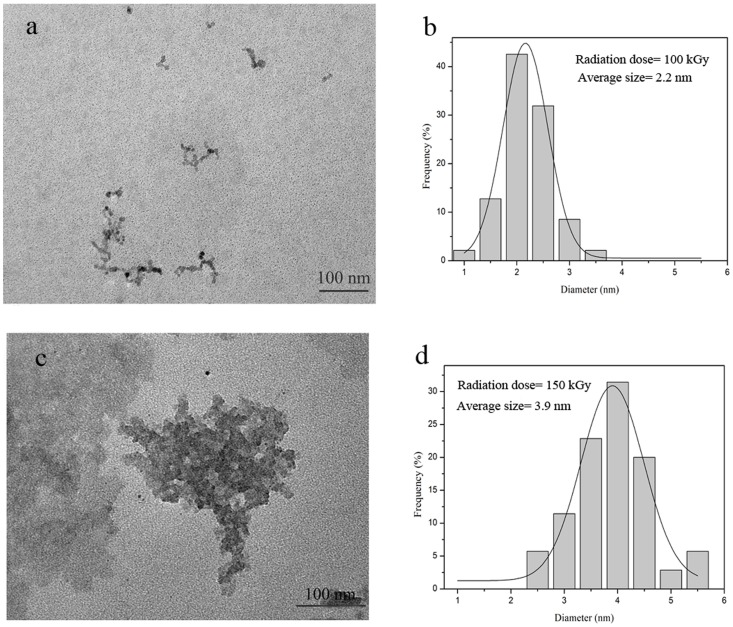
TEM images and size distributions of Fe_3_O_4_ nanoparticles synthesized at: **a, b**) 100 kGy, with average size of 2.2 nm, and **c, d**) 150 kGy, with average size of 3.9 nm.

Figure 2 shows the TEM results for the iron oxide nanoparticles obtained at the 100 kGy and 150 kGy radiation doses and their associated size distribution. It was observed that the nanoparticles in all of the samples are generally spherical in the shape, and their average size increases with the dose, from 2.2 nm at 100 kGy to 3.9 nm at 150 kGy. Increasing the radiation dose favored the generation of more solvated electrons, which in turn enhanced the reduction rate and, consequently, increased the Fe^2+^/Fe^3+^ ratio. This enhanced ratio leads to a greater number of resulting nanoparticles. These small particles collide with each other according to their random movement in the solution, and the magnetic interactions between these nanoparticles leads to particle aggregation. Figure 2-c clearly shows that the agglomeration of particles occurred in a sample irradiated at a higher dose. By increasing the gamma dose from 150 to 200 kGy, nanoparticles have been surrounded by a thicker layer of PVA as a result of crosslinking of the polymer chains. Because of this crosslinking phenomenon the TEM image cannot be formed clearly in this range of radiation (Figure S1).

**Figure 3:**
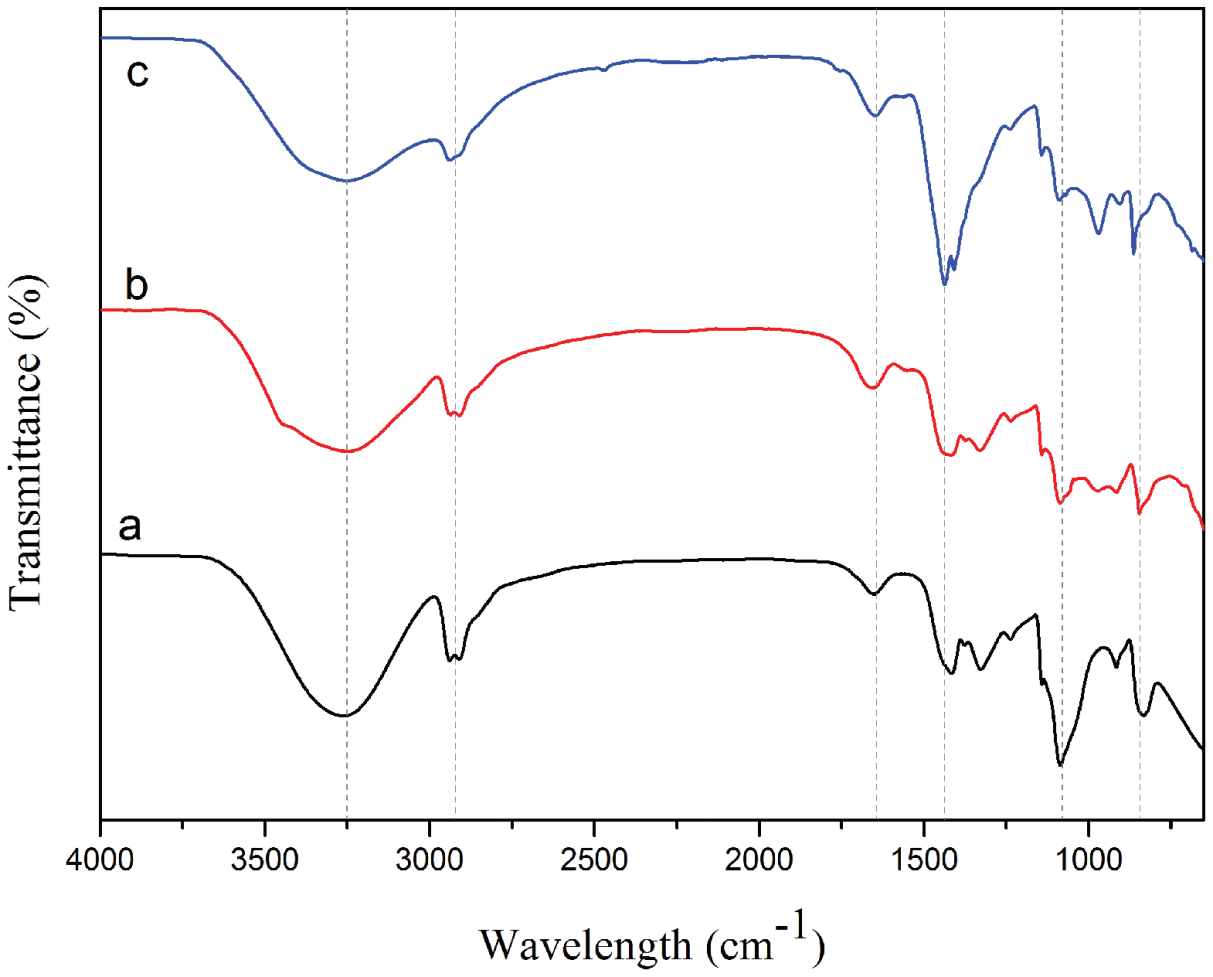
FTIR spectra of samples before and after irradiation. (**a**) PVA-iron ion complexes before irradiation and irradiated PVA- iron oxide nanoparticles at (**b**) 100 kGy, and (**c**) 150 kGy gamma doses.

The presence of specific chemical groups in the materials and attachment of the polymer on to the surface of nanoparticles have been investigated using FTIR. Figure 3 shows the FTIR spectra of the PVA-Fe^3+^ complexes before irradiation and coated iron oxide nanoparticles after irradiation at different doses. The strong broad band observed around 3,300 cm^−1^ has been assigned to the stretching of alcoholic O–H groups resulting from the intra- and inter-molecular type of hydrogen bonds in the polymer. A considerable reduction in the intensity and an increase in the width of the O–H peaks with increasing gamma dose indicates the possible formation of crosslinked polymer chains under irradiation [30]. C–H stretching vibrations were observed at 2,930 cm^−1^ [31]. The peaks at 1,700 and 1,450 cm^−1^ correspond to C = O and asymmetrical COO^−^ stretching vibrations, respectively [31], [32], [33]. These vibrational bands confirm coordination of the metal oxides by the carboxylate groups of the polymer. The additional band around 1,100 cm^−1^, which appeared at all doses, corresponds to the C–O stretching peak in the presence of iron ions in the resultant polymeric complexes [34]. The small peak present at 850 cm^−1^ is attributed to CH_2_ stretching vibrations which is observed in the PVA-coated iron oxide particles [33].

### Effect of radiation dose on structure and magnetic properties

**Figure 4:**
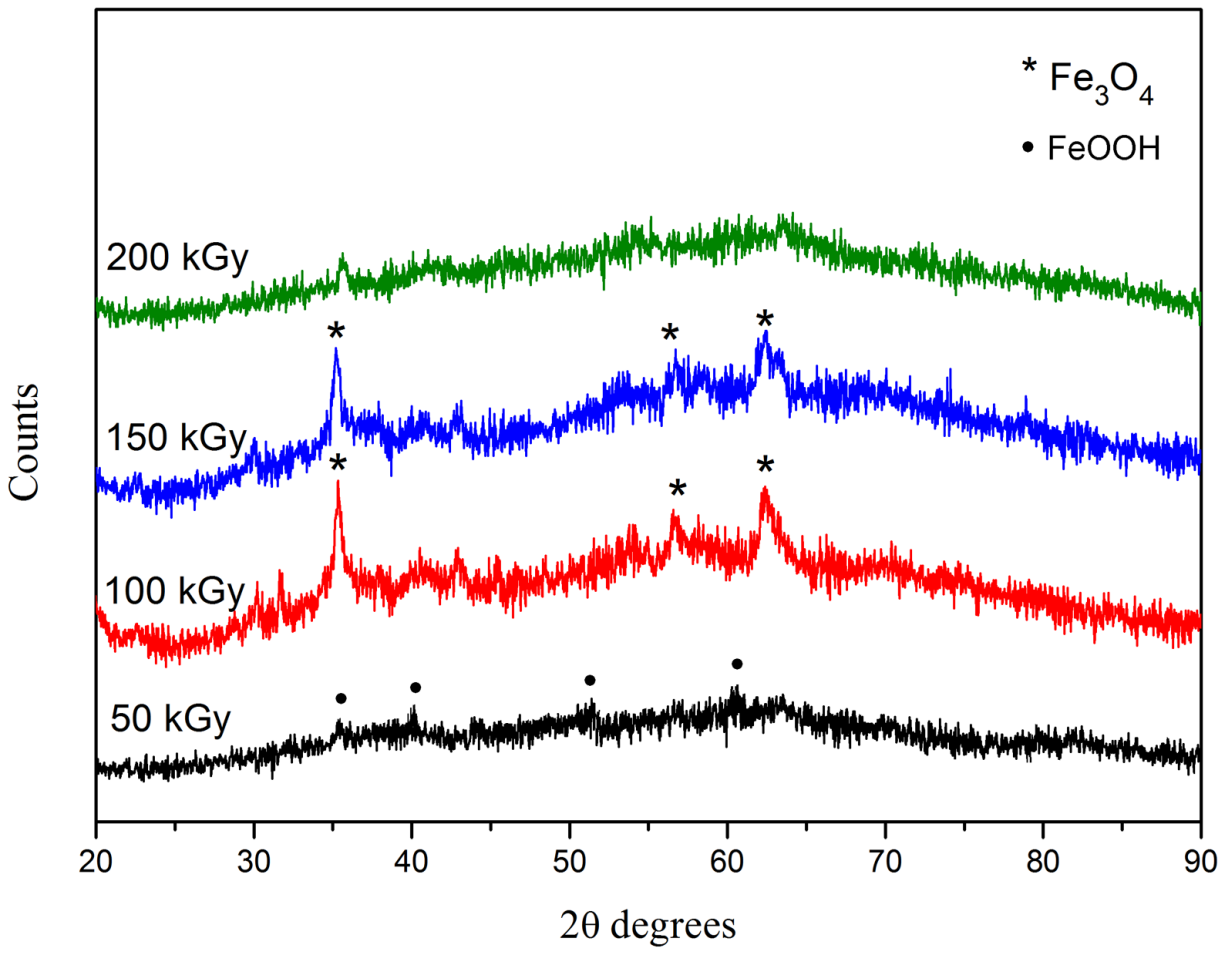
X-ray diffraction patterns of iron oxide nanoparticles at different radiation doses.

Increasing the radiation dose can increase the Fe^2+^/Fe^3+^ ratio. Small values of this ratio (<0.3) are known to produce goethite (α-FeOOH) preferentially, especially in wet environments [35]. At low doses (<100 kGy), because of the lack of solvated electrons, the Fe^2+^ ion concentration was lower than the concentration of unreduced Fe^3+^ ions. This condition favors the formation of poorly crystallized phases of α-FeOOH as demonstrated by XRD (Figure 4). By increasing the gamma dose, the Fe^2+^/Fe^3+^ ratio increases, and the resultant particles become more homogenous in composition with higher degree of ordering [35], [36]. The lattice constants, calculated from the XRD patterns in Figure 4, were 8.41 Å for the sample irradiated at 100 kGy, and 8.4 Å for the sample irradiated at 150 kGy. These values are closer to that of Fe_3_O_4_ (8.396 Å) than γ-Fe_2_O_3_ (8.345 Å) [4]. This result indicates that, by increasing the dose, the Fe_3_O_4_ phase becomes the preferred phase. The intensity of the Fe_3_O_4_ peaks decreases with increasing radiation dose and disappears at 200 kGy. This might be related to the increase in the number of nanoparticles in the amorphous phase, which is as a result of greater crosslinking in the PVA coatings [37].

The magnetic properties of nanoparticles are determined by many factors, including their chemical composition, the type and the degree of defectiveness of the crystal lattice, the global electron configuration of molecules, the interaction between neighboring spins, and the particle size and shape [38].

**Figure 5:**
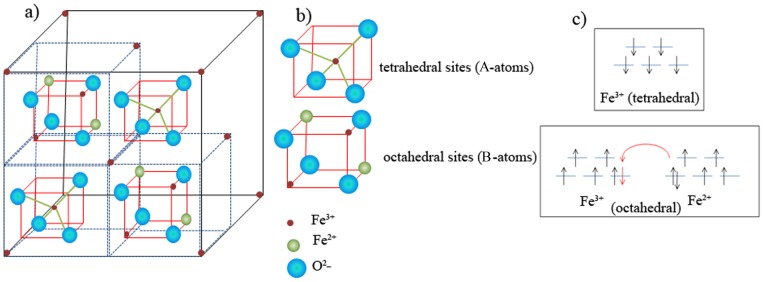
Schematic figures of : (**a**) Simplified crystal structure of Fe_3_O_4_; (**b**) Octahedral and tetrahedral sites showing cations and oxygen; (**c**) Schematic depiction of the splitting of 5 d orbitals of iron ions in octahedral and tetrahedral sites.

Magnetite has an inverse spinel structure with a mixed (Fe^2+^ and Fe^3+^) iron oxide content composed with a cubic close-packed oxygen array [39]. Its formula is written as Fe^3+^_A_[Fe^3+^Fe^2+^]_B_O_4_, and in its unit cell, all tetrahedral sites are occupied by Fe^3+^ (“A” sites), and octahedral sites are occupied by both Fe^3+^ and Fe^2+^ (“B” sites) (Figure 5-a and 5-b) [40]. The electron spins in the Fe^2+^ and Fe^3+^ ions in the octahedral sites are coupled, and the spins of the Fe^3+^ ions in the tetrahedral sites are coupled as well, but anti-parallel to the former (Figure 5-c). As can be seen in Figure 5-c, Fe^3+^ and Fe^2+^ ions with octahedral coordination are ferromagnetically coupled through a double-exchange mechanism. An electron, whose spin is colored red, can be exchanged between two octahedral sites. On the other hand, the Fe^3+^ ions in tetrahedral and octahedral sites are coupled anti-ferromagnetically via the oxygen atom. In this configuration, all Fe^3+^ spins cancel each other out and thus the unpaired spins of Fe^2+^ ions in octahedral sites contribute to the overall magnetization. The multiple magnetic domains of Fe_3_O_4_ exhibit magnetic moments, which are all aligned within a given domain, but between the domains magnetic moments are oriented in random directions. Thus, it can be categorized as a ferrimagnetic material [41].

Decreasing the size of the magnetic body results in two important types of magnetic behavior, namely, single-domain ferrimagnetism and superparamagnetism. In single-domain nanoparticles, only coherent magnetization rotation can overcome the effective anisotropy of the particle, but domain wall movement is not possible. By decreasing the particle size, the magnetization is no longer stable and the particle is said to be superparamagnetic [42].

**Figure 6:**
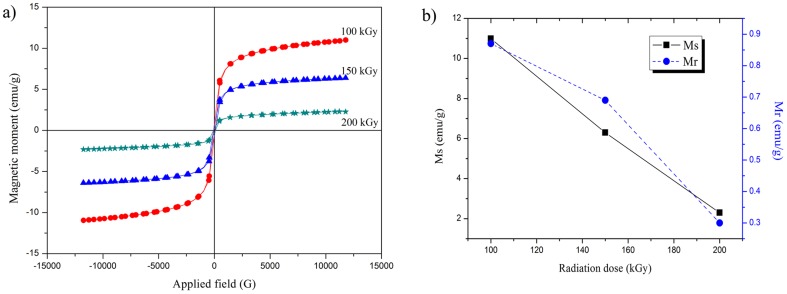
Magnetic properties of synthesized magnetite nanoparticles at room temperature. (**a**) Hysteresis loop of magnetite nanoparticles at various radiation doses; (**b**) Variation of *Ms*(emu/g) and *Mr*(emu/g) vs. radiation dose.

The superparamagnetic behavior of our nanoparticles fabricated using various radiation doses are illustrated by the hysteresis loops collected at 300 K, as shown in Figure 6-a. At room temperature, all irradiated samples exhibit negligible magnetic remanence, and the initial slopes of the magnetization curves are steep. These facts are related to finite-size and surface effects in the superparamagnetic nanoparticles [43]. The variation in saturation magnetization and remanence with radiation dose are shown in Figure 6-b. It is evident that, when the radiation dose increases from 100 to 200 kGy, the saturation magnetization decreases from 10.99 to 2.3 emu/g. The largest saturation magnetization was 10.99 emu/g for the sample irradiated at 100 kGy, which is much lower than that reported for the bulk magnetite (92emu/g). The decrease in size, from bulk to nano scope, causes the large surface spin canting, and consequently, a significant reduction in the magnetization value [44].

In addition, the appearance of the polymer layer on the outer shells of magnetite particles can reduce the saturation magnetization. This can be interpreted as a reduction of the magnetic volume. The magnetic moments of the surface Fe_3_O_4_ particles bonded to carbonyl chain of polymer and therefore being quenched [45]. The variation in the saturation magnetization of the samples with increasing dose can be attributed to the existence of a magnetically disordered spin layer on the particles' surface, or the existence of canted spins [46]. On the other hand, with increasing radiation dose the thickness of the polymer coating on the surface of nanoparticles increases because of the higher degree of crosslinking. This results in decreased values of the saturation magnetization. The dependence of remanence on radiation dose is shown in Figure 6-b. Decreases in the inter-grain exchange coupling force resulting from increases in size, leads to randomization of the orientation. This behavior has been associated with thick polymer layers produced by high radiation doses and leads to smaller remanence values [47].

**Figure 7:**
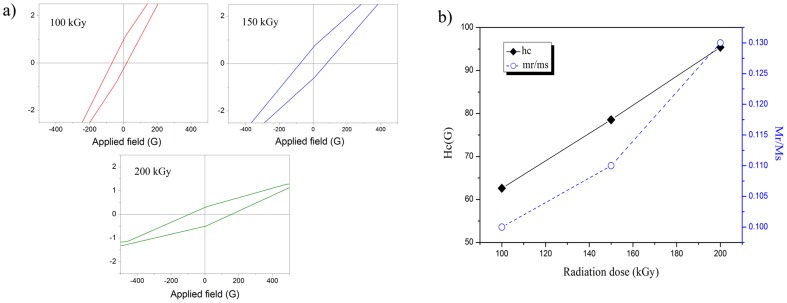
Effect of radiation dose on magnetic properties of resultant nanoparticles. (**a**) Hysteresis loops under a low applied field and (**b**) variation of the coercivity field (*H_c_*) and remanence ratio (*R = Mr/Ms*) with the radiation dose.

In finite-size nanoparticles, which have no mobile domain walls, the magnetization will be reversed through spin rotation rather than through the motion of domain walls. This results in larger coercivity of the nanoparticles compared to their bulk counterparts [46]. Figures 7-a and 7-b show that the values of the coercivity field (*H_c_*) and remanence ratio (*R = Mr/Ms*) of the Fe_3_O_4_ nanoparticles increase with increasing gamma dose from 100 to 200 kGy as a result of particle size enhancement. Another reason for the increased coercivity with radiation dose is the strong bonds established between adsorbed crosslinked polymer layers on the surface of iron oxide nanoparticles, which could block spin-flip conversion. The remanence ratio, which is described as the existence or absence of different types of inter-grain group exchanges, varies from 0 to 1 [48]. Values of *R* lower than 0.5 indicate that the particles interact by magnetostatic interactions, while *R* = 0.5 indicates randomly oriented non-interacting particles that undergo coherent rotations. The observed values between 0.5 and 1 confirm the existence of exchange-coupling particles [49]. Therefore, the *R* values for samples irradiated at 100, 150, and 200 kGy are lower than 0.5 are attributed to particles that interact by magnetostatic interactions.

## Conclusion

In summary, magnetite nanoparticles with different sizes and crystallinities have been synthesized using radiolytic reduction method by gamma irradiation. The XRD studies confirm the cubic crystal structure of Fe_3_O_4_, especially at the 100 kGy dose. The TEM images indicate that the synthesized magnetite nanoparticles are almost spherical in shape and that the particle size increases with absorbed dose. An FT-IR analysis of the colloidal nanoparticles confirmed that PVA was absorbed onto the outer shell of Fe_3_O_4_ nanoparticles during particle growth. All samples exhibit superparamagnetism, and their saturation magnetization, remanence and coercivities were found to be dependent on the particle size, crystallinity, and the thickness of adsorbed crosslinked polymer layers on the surface of iron oxide nanoparticles.

## Supporting Information

Figure S1 **TEM image of Fe_3_O_4_ nanoparticles synthesized at 200 kGy.** This image cannot be formed clearly in higher magnification because of thick layer of crosslinked polymer coating.
(TIF)
