# Supplementary figures and images for: Radiolytic Formation of Fe3O4 Nanoparticles: Influence of Radiation Dose on Structure and Magnetic Properties
Source: PLoS One. 2014 Mar 7;9(3):e90055. doi: 10.1371/journal.pone.0090055 (PMC3946508; doi:10.1371/journal.pone.0090055)

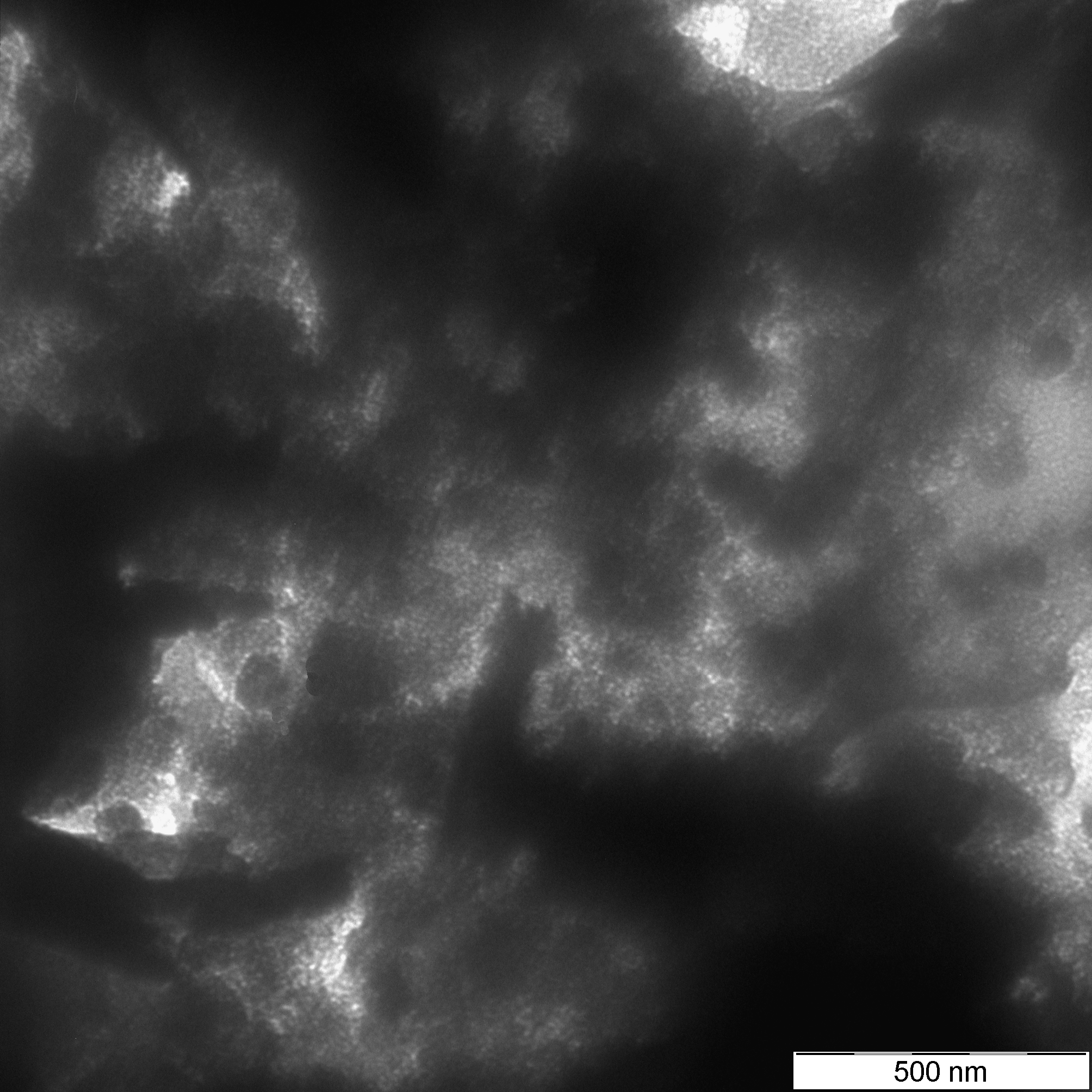

Supplement: Figure S1 — TEM image of Fe3O4 nanoparticles synthesized at 200 kGy. This image cannot be formed clearly in higher magnification because of thick layer of crosslinked polymer coating. (TIF) [file pone.0090055.s001.tif]
